# TCN-LSTM-AM Short-Term Photovoltaic Power Forecasting Model Based on Improved Feature Selection and APO

**DOI:** 10.3390/s25247607

**Published:** 2025-12-15

**Authors:** Ning Ye, Chaoyang Zhi, Yongchao Yu, Sen Lin, Fengxian Liu

**Affiliations:** School of Automation and Electrical Engineering, Shenyang Ligong University, Shenyang 110159, China; yening_neu@sylu.edu.cn (N.Y.); 2406620673@stu.sylu.edu.cn (Y.Y.); linsen@sylu.edu.cn (S.L.); lfx9488@sylu.edu.cn (F.L.)

**Keywords:** solar power forecasting, deep learning, machine learning, APO algorithm

## Abstract

The inherent volatility and intermittency of solar power generation pose significant challenges to the stability of power systems. Consequently, high-precision power forecasting is critical for mitigating these impacts and ensuring reliable operation. This paper proposes a framework for photovoltaic (PV) power forecasting that integrates refined feature engineering with deep learning models in a two-stage approach. In the feature engineering stage, a KNN-PCC-SHAP method is constructed. This method is initiated with the KNN algorithm, which is used to identify anomalous samples and perform data interpolation. PCC is then used to screen linearly correlated features. Finally, the SHAP value is used to quantitatively analyze the nonlinear contributions and interaction effects of each feature, thereby forming an optimal feature subset with higher information density. In the modeling stage, a TCN-LSTM-AM combined forecasting model is constructed to collaboratively capture the local details, long-term dependencies, and key timing features of the PV power sequence. The APO algorithm is utilized for the adaptive optimization of the crucial configuration parameters within the model. Experiments based on real PV power plants and public data show that the framework outperforms multiple comparison models in terms of key indicators such as RMSE (2.1098 kW), MAE (1.1073 kW), and R^2^ (0.9775), verifying that the deep integration of refined feature engineering and deep learning models is an effective way to improve the accuracy of PV power prediction.

## 1. Introduction

Against the backdrop of increasingly severe global environmental challenges and finite fossil fuel resources, developing clean energy has become a shared global solution for addressing ecological and environmental issues while achieving sustainable social development [1]. Renewable energy sources, exemplified by photovoltaics, are advancing at an unprecedented pace. However, the inherent intermittency and variability of PV generation complicate the stable operation of power systems and hinder effective dispatch scheduling [2]. High-precision PV power forecasting technology is crucial for addressing the aforementioned challenges and enhancing the grid’s capacity to accommodate new energy sources [3,4].

Current short-term PV power forecasting models are primarily categorized into physical models, statistical models, and machine learning models [5,6,7]. Physical models employ complex equations to simulate photovoltaic systems; these models are computationally expensive and fail to capture all the details of real-world conditions, resulting in inaccurate predictions [8]. While grounded in historical records and mathematical formulations, statistical approaches often lack the sensitivity to capture transient meteorological anomalies, which are critical determinants of photovoltaic output [9]. Machine learning models achieve higher levels of accuracy by leveraging real-time data, integrating historical models, and accommodating evolving conditions. They are adept at capturing relationships among nonlinear patterns in PV data [10]. Consequently, to enhance the precision of PV power forecasting, efforts primarily focus on two aspects: feature extraction and model construction [11].

The quality of raw data and feature engineering design are crucial for enhancing the accuracy of solar power forecasting [12]. Not all input features are relevant to the prediction target; some features may contain noise, thereby degrading prediction performance [13]. Therefore, the objective of feature selection is to identify a feature subset with high information content, thereby improving model prediction accuracy while minimizing computational complexity [14]. In real-world engineering scenarios, data loss caused by sensor faults or communication disruptions is quite common. Traditional gap-filling methods, such as mean interpolation and linear interpolation, are easy to implement but often result in significant bias when handling photovoltaic data with strong periodic and nonlinear characteristics. These approaches may distort the intrinsic statistical distribution of the dataset. In recent years, data-driven interpolation techniques based on machine learning have attracted increasing interest. Among them, the k-nearest neighbor algorithm is widely used because it can utilize the correlation between multiple variables and has shown strong performance in reconstructing nonlinear time-series data. For feature screening, Pearson’s correlation coefficient (PCC) is frequently employed to identify features with strong linear relationships. However, PV power generation involves highly coupled multi-physics processes, in which meteorological factors and power generation interact in a complex nonlinear manner—interactions that cannot be adequately captured by linear indicators such as PCC. Reference [15] proposes an advanced deep learning interpolation network. It emphasizes the importance of capturing complex nonlinear relationships for prediction accuracy and offers a more sophisticated solution approach based on neural networks. The game-theoretic SHAP method provides a deeper perspective by quantifying the marginal contribution of each feature to the model’s predictions, thereby effectively identifying key nonlinear influencing factors. Reference [16] quantifies the impact of different input features on model outputs using the SHAP method based on symmetric decision tree models, focusing on feature importance. Reference [17] employs SHAP values to explicitly quantify the marginal contributions of features to model outputs, thereby measuring global importance and analyzing the positive or negative correlation between features and PV power.

Early studies in model construction mostly used statistical models, such as ARIMA. These models do not capture the nonlinear characteristics of photovoltaic sequences very well [18]. The current focus of PV power generation prediction research is on prediction technology based on deep learning, which is developed from artificial neural networks (ANNs) [19]. Neural networks, represented by Long Short-Term Memory (LSTM) and Convolutional Neural Networks (CNNs), are widely applied due to their advantages in processing time-series data. Reference [20] integrates a CNN with nonlinear quantile regression (QR) to extract nonlinear features and construct nonlinear QR functions. Consequently, the enhanced CNN efficiently handles complex data, while the QR model provides prediction information for PV power.

The DSCLANet introduced in [21] integrates a self-attention mechanism into a dual-stream CNN-LSTM architecture, a design that successfully yielded favorable prediction outcomes. Reference [22] proposes a PV power forecasting model founded upon an enhanced VMD and LSTM network. By analyzing factors influencing PV power and incorporating decomposed power sequences and meteorological factors as model inputs, power forecasting is performed. Simulation results show this model is more accurate than BPNN and LSTM, providing a reference for power forecasting in PV power plants. Reference [23] presents an integrated multivariate model for PV power forecasting based on VMD, CNNs, and Bidirectional Gated Recurrent Units (BiGRU). PV power sequences are decomposed into multiple sub-modes via VMD, combined with meteorological data for feature extraction and prediction, and finally integrated to obtain overall power forecasts. Research indicates that this model outperforms univariate models when incorporating meteorological variables and demonstrates significant predictive advantages over other methods. Reference [24] combines particle swarm optimization, variational mode decomposition, convolutional neural networks, and an embedded attention mechanism to decompose raw power load sequences, with the attention mechanism enhancing prediction accuracy. To mitigate the effects of large-scale wind power systems on the electrical grid and enhance the exploitation of wind power, Reference [25] proposes a method of decomposing wind speed using CEEMDAN, demonstrating the efficacy of the method. Reference [26] introduces a hyper-short-term PV power forecasting model fully integrating CEEMDAN with RIME optimization and AM-TCN-BiLSTM, further improving PV prediction accuracy. Reference [27] proposed a short-term PV power forecasting model based on weather, employing AHA-VMD-MPE and an improved ensemble approach for decomposing and reconstructing data. While validated using measured data from multiple PV plants, the analysis of data characteristics under specific weather types was somewhat limited. Reference [28] incorporated a universal physical model of PV systems into machine learning, using a Danish PV system as a basis for the study. Multiple machine learning methods were applied to validate the effectiveness of the proposed approach. Table 1 below records the data resolution, evaluation metrics, and main contributions and shortcomings of more advanced models in recent years.

Additionally, metaheuristic algorithms are frequently employed to predict relevant model parameters. Examples include genetic algorithms [36], particle swarm optimization [37], and the multi-objective gray wolf optimizer [38]. These algorithms can also improve the accuracy of forecasts for critical time sequences. Reference [39] utilizes PSO to optimize the CNN-LSTM model, improving its adaptability and prediction accuracy. Reference [40] established an MBES-LSTM model based on the improved Harrier algorithm, which improved the accuracy of wind power prediction. Reference [41] optimized GRU-ATT hyperparameters using an improved CPO algorithm, yielding significantly better prediction results than baseline models. However, employing optimization algorithms for model parameter prediction requires additional data training processes, which slows model training and may induce overfitting, negatively impacting predictive performance [42]. Consequently, model parameter tuning should use optimization algorithms that converge quickly and are highly stable.

Despite some progress in existing research, the following problems remain:

(1) Most works only improve single aspects of data preprocessing or model structure, lacking a systematic design for synergistic optimization of both.
(2) Current feature selection methods largely rely on linear correlation analysis, which struggles to characterize the complex nonlinear coupling relationships between meteorological factors, leading to the neglect of some crucial information.
(3) The performance of models is highly sensitive to hyperparameter settings, while common parameter tuning methods are inefficient and fail to achieve global optima, thus preventing the full realization of the model’s potential.

To address the above challenges, this paper proposes a two-stage PV power generation forecasting framework that deeply integrates refined data processing with advanced forecasting models. Its main contributions are as follows:

(1) Unlike traditional methods that rely solely on statistical correlation for feature selection, this paper proposes a KNN-PCC-SHAP feature engineering method. This method effectively handles missing data through a coarse-screening and fine-tuning strategy, and introduces SHAP value analysis based on cooperative game theory to quantify the marginal contribution of each feature to the result. By comprehensively considering the linear correlation and nonlinear effects of features, a more information-concentrated feature subset is constructed.
(2) To address the complex temporal characteristics of photovoltaic data, a TCN-LSTM-AM prediction model is constructed. This model utilizes TCN expanded causal convolution to enlarge the receptive field for extracting local features of the sequence, combines LSTM to learn long-term dependencies, and introduces an attention mechanism (AM) to focus on key information, achieving a balance between prediction accuracy and computational efficiency.
(3) To address the inefficiency of conventional parameter tuning methods and their inability to bring out the potential of models, we introduce the APO algorithm for adaptive hyperparameter optimization. This algorithm simulates the global exploration and local exploitation behaviors of biological organisms, achieving a balance between exploration and exploitation, thereby further enhancing the model’s predictive capabilities.

## 2. Materials and Methods

**Figure 1:**
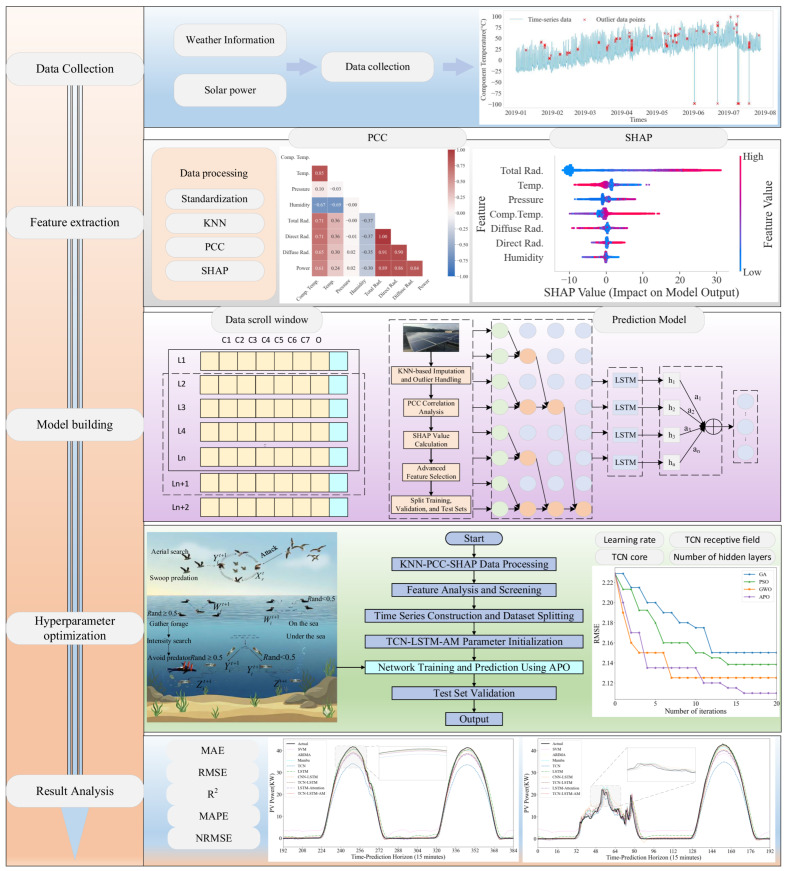
Overall Article Framework Diagram.

The PV power forecasting framework presented in this paper consists of two main stages: data processing and deep learning-based power prediction. As illustrated in Figure 1, this overall framework aims to achieve high-accuracy PV power generation forecasting by systematically improving data and constructing advanced models.

### 2.1. Data Processing and Feature Engineering

#### 2.1.1. Missing Value Imputation Based on KNN

The integrity of collected datasets in solar farms is often compromised by equipment-related constraints, specifically sensor outages and transmission instability. Traditional methods like mean or linear interpolation, though simple, can distort the original distribution characteristics of photovoltaic data with strong periodicity and nonlinear features, thereby introducing significant deviations. To mitigate the impact of such data loss, we leverage the KNN technique to reconstruct the missing entries. The core idea is that the missing value of a sample can be estimated using the known values of its K most similar neighbors in a multi-dimensional feature space [43]. The advantage of KNN lies in its independence from assumptions about the overall data distribution, effectively leveraging information from multiple relevant features to infer missing values. Consequently, these attributes render the model exceptionally well suited for processing photovoltaic data characterized by intricate patterns.

#### 2.1.2. Preliminary Feature Selection Based on PCC

To reduce both computational complexity and the potential for overfitting in subsequent models, the PCC is first employed for preliminary feature screening after data imputation. The PCC is an indicator that measures the linear correlation of two continuous variables [44] and is calculated using Equation (1).

(1) $$\rho_{XY}=\frac{\sum_{i=1}^{n}\left(X_{i}-\bar{X}\right)(Y_{i}-\bar{Y})}{\sqrt{\sum_{i=1}^{n}{\left(X_{i}-\bar{X}\right)}^{2}}\sqrt{\sum_{i=1}^{n}{\left(Y_{i}-\bar{Y}\right)}^{2}}}$$

Here, *X* and *Y* represent the feature variable and target variable, respectively, while $\bar{X}$ and $\bar{Y}$ are their mean values. By calculating the absolute value of the PCC between each meteorological feature and the PV power output, we can quickly identify and retain features with strong linear correlations while eliminating those that are linearly irrelevant or weakly correlated, thereby achieving preliminary dimensionality reduction of the feature space.

#### 2.1.3. Deep Feature Selection Based on SHAP

However, PCC can only capture linear relationships and fails to reveal complex nonlinear influences. This paper introduces SHAP as a means of addressing this limitation through refined second-stage feature selection. The theoretical foundation of SHAP lies in cooperative game theory, serving as a powerful tool for model interpretability [45]. It quantifies the marginal contribution of individual features to prediction outcomes, known as SHAP values. The SHAP value $\phi_{j}$ for feature j is calculated as the average contribution to the model of introducing feature j across all possible subsets of features, as shown in Formula (2):

(2) $$\phi_{j}=\sum_{S\subseteq N\setminus \{j\}}\frac{|S|!(|N|-|S|-1)!}{|N|!}$$

where N is the total set of features, S is a subset of N that excludes feature j, and v(S) is the model’s predictive output when using only the feature subset S. The benefit of SHAP values stems from their ability to account for the direct influence of features and to capture interaction effects between features. A high SHAP value for a feature indicates its stable and significant contribution to the model’s predictive outcomes.

**Figure 2:**
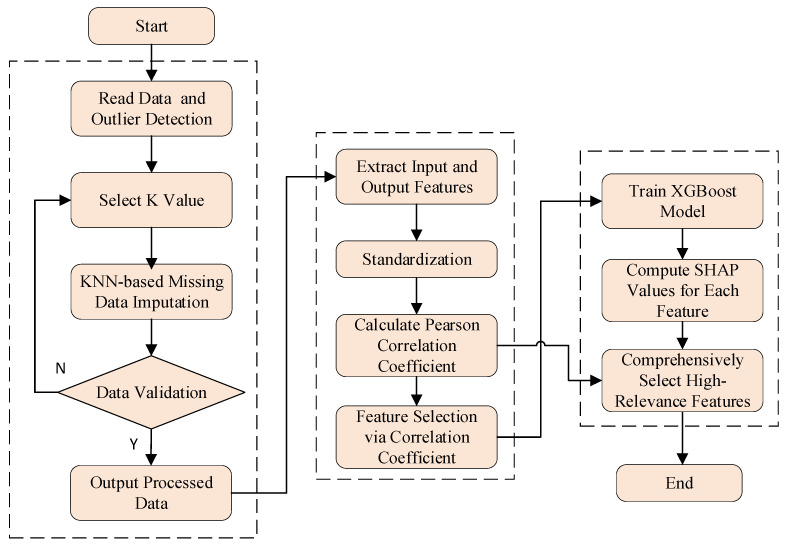
Feature processing flowchart.

The specific steps are illustrated in Figure 2. In this framework, we first train a high-performance XGBoost gradient boosting tree model using the feature set preliminarily screened by PCC. Subsequently, this model is utilized to compute SHAP values for all samples in the dataset, and the global importance of each feature is summarized through aggregation. Finally, based on this importance ranking, the top-ranked features are selected as the optimal feature subset to be fed into the predictive model. This process ensures that the selected features are those that truly serve as key drivers within the complex nonlinear model.

### 2.2. TCN-LSTM-AM Combined Forecasting Model

#### 2.2.1. TCN Network

**Figure 3:**
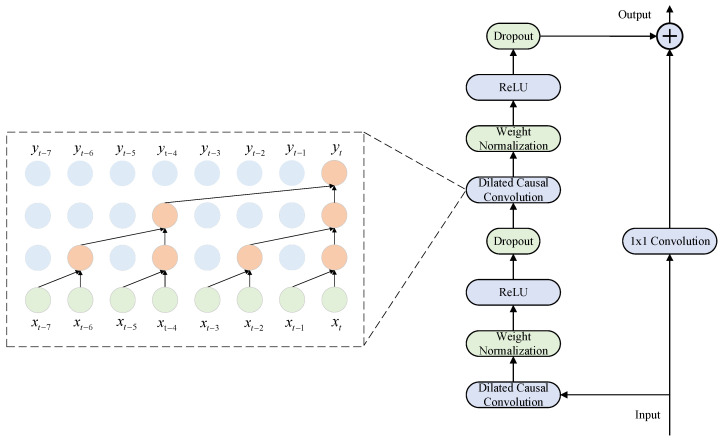
TCN Network Core Architecture Diagram.

The TCN network is a convolutional architecture specifically designed for sequential data [46]. The structure diagram is shown in Figure 3. The primary objective is to prevent any leakage of future information in predictions through causal convolutions, while exponentially scaling the receptive field at relatively low computational cost via dilated convolutions. This approach effectively captures both long-range local patterns and short-term fluctuating information. To avoid inflating the hidden layer dimensions while still capturing extended time-series patterns, the model leverages dilated convolution alongside causal filters, effectively broadening the receptive field, as shown in Equation (3).

(3) $$F(m)=(x⊗f_{d})(m)=\sum_{i=0}^{k-1}f(i)\cdot x_{m-d-i}$$

In the formula, k is the size of the convolution kernel, x is the input sequence, m is the sequence element, d is the dilation rate, and ⊗ represents the convolution operation.

Residual connections enable cross-layer information transfer by establishing skip connections between non-adjacent layers. This mechanism incorporates input data from the previous layer’s transposed causal convolutional layer into the output of the next or deeper layer, thereby enhancing the network model’s generalization capability. First, perform one-dimensional dilated convolutions, then normalize the weights and use the ReLU function as the activation function. Within this framework, the TCN module serves as an efficient feature encoder, responsible for extracting refined deep feature representations from multidimensional input sequences.

#### 2.2.2. Introduction to LSTM Networks

**Figure 4:**
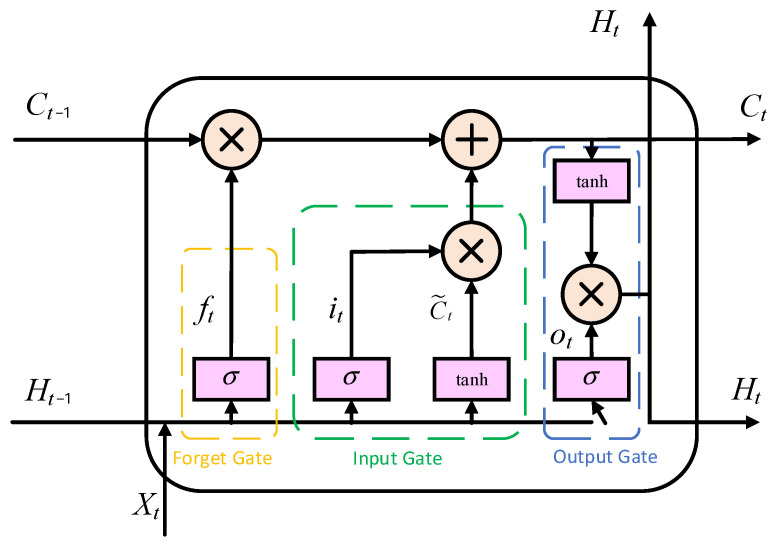
LSTM Neural Network Structure.

LSTM, as a special type of recurrent neural network, is designed to address the long-term dependency issues inherent in traditional RNNs [47]. The gating mechanism of LSTM includes the forget gate, the input gate, and the output gate. Through its sophisticated internal gating mechanism, it can selectively remember and forget information, making it highly suitable for processing and predicting long time-series data. Figure 4 shows the LSTM unit.

#### 2.2.3. Attention Mechanism

**Figure 5:**
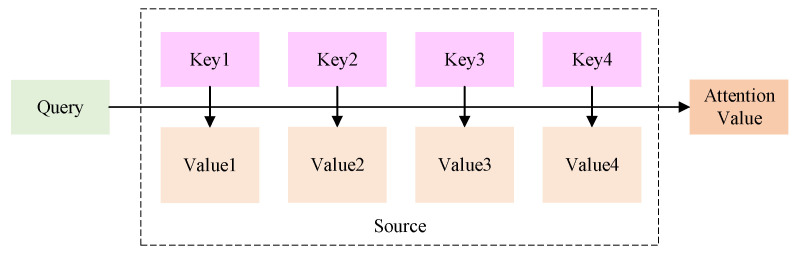
Attention Function Diagram.

When processing long sequences of data, RNNs may progressively reduce the weight assigned to information from previous time points in the neural state. This can make it difficult for the network to capture features from previous time points, resulting in information being overlooked. Attention mechanisms mimic the way the human brain focuses on critical information. This enhances its contribution to network training, thereby improving the accuracy of the training process. Attention mechanisms enable models to focus more on critical factors by assigning varying weights to input features, thereby making more accurate predictions. We can describe its function as a way of mapping from a query to a set of key-value pairs [48,49]. A schematic representation of the attention function is shown in Figure 5.

First, use the similarity function to calculate the similarity with each element, thereby obtaining the weight. The formula is shown in (4):

(4) $$f(Q_{t},K_{s})=\left\{\begin{array}{ll} Q_{t}^{T}K_{s} & dot\\ Q_{t}^{T}W_{a}K_{s} & general\\ arg[Q_{t}:K_{s}] & concat\\ v_{a}^{T}\tanh(W_{a}Q_{t}+U_{a}K_{s}) & perceptron \end{array}\right.$$

$Q_{i}$ represents the target module, and $K_{s}$ represents the source module. $W_{a},U_{a},v_{a}$ are all weight matrices or vectors learned during the model training process, which are used for linear transformation. dot directly calculates the dot product of $K_{s}$ and $Q_{t}$. general introduces a weight matrix $W_{a}$ on the basis of the dot product. concat is usually used in combination with a perceptron. It concatenates $Q_{t}$ and $K_{s}$ into a longer vector and then inputs it into a feed-forward neural network, perceptron passes $Q_{t}$ and $K_{s}$ through different linear layers $W_{a},U_{a}$ respectively, then adds them together and passes through the tanh activation function, and finally maps them to a scalar score through a linear layer $v_{a}^{T}$. Secondly, use the softmax function for normalization to obtain the attention weights, as shown in Equation (5):

(5) $$a_{t}=\mathrm{softmax}\left(f(Q,k_{i})\right)=\frac{exp\left(f(Q,k_{i})\right)}{\sum_{j}exp\left(f(Q,k_{j})\right)}$$

Finally, perform a weighted sum of the normalized weights and the corresponding value, and obtain the final value, as shown in (6).

(6) $$\mathrm{Attention}(Q,K,V)=\Sigma_{i}a_{i}V_{i}$$

#### 2.2.4. Establishment of TCN-LSTM-AM Model Establishment

**Figure 6:**
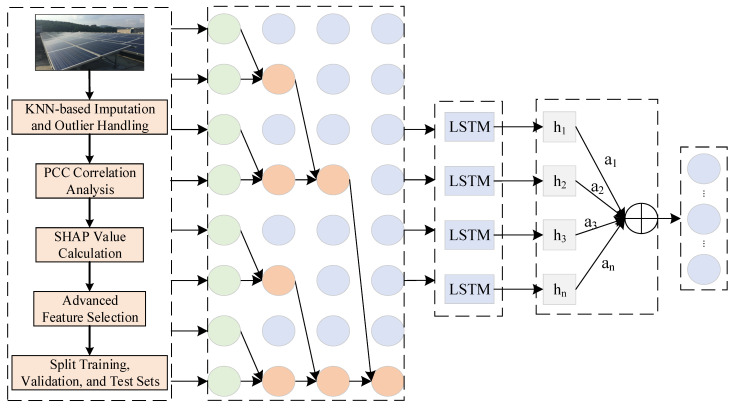
TCN-LSTM-AM Model.

Figure 6 shows the architecture of the TCN-LSTM-AM model proposed in the article. The specific data processing flow is as follows.

First, the multi-dimensional input sequence after feature engineering is fed into the TCN network. As a feature extractor, TCN is responsible for capturing fine local patterns and short-term fluctuation information from the original sequence.

Then, the output of the TCN network will be input into the LSTM network as a feature sequence. The LSTM network further processes these features to learn and remember the sequence’s underlying long-term dependencies.

At the output of the LSTM, an attention mechanism is applied to weight the features at various time steps and emphasize the essential information in the prediction results. These weighted features are ultimately fed into a fully connected layer to produce the PV power prediction.

### 2.3. Arctic Puffin Optimization (APO) Algorithm

A new metaheuristic algorithm called APO has been developed to simulate the biological behavior of Atlantic puffins [50]. It employs mathematical models to emulate two core behaviors of puffins: aerial flight and underwater foraging. This bio-inspired simulation enables the APO algorithm to find the right balance between global exploration and local exploitation. This gives it strong global optimization capabilities and the ability to avoid falling into local optima. This computational framework primarily encompasses population initialization, the aerial flight stage, and the underwater foraging stage, alongside a transition strategy to alternate between these behaviors.

#### 2.3.1. Population Initialization

Every Arctic puffin represents a potential solution in the solution space. During the initialization phase, a population containing N individuals is randomly generated within a given range, as shown in Equation (7).

(7) $$\vec{X_{i}^{t}}=rand\times (ub-lb)+lb,i=1,2,3\ldots N$$

Here, ${\vec{X}}_{i}^{t}$ is the position of the i-th puffin; rand is a random number between 0 and 1; lb and ub denote the upper and lower bounds of the interval, and N is the size of the population.

#### 2.3.2. Aerial Flight Phase

This phase simulates the aerial reconnaissance and hunting behavior of puffins, focusing on global search to explore a wider solution space. It includes two main strategies.

The aerial search strategy simulates the coordinated flight of puffins to scout prey, introducing the Levy flight mechanism to enhance random exploration capabilities. The individual position update process is shown in Equations (8)–(10).

(8) $$\vec{Y_{i}^{t+1}}=\vec{X_{i}^{t}}+\left(\vec{X_{i}^{t}}-\vec{X_{r}^{t}}\right)*L(D)+R$$

(9) R=round(0.5∗(0.05+rand))∗α

(10) α~Normal(0,1)

where r is an integer between 1 and N, $\vec{X_{i}^{t}}$ represents the current candidate solution; $\vec{X_{i}^{r}}$ represents a candidate solution randomly selected from the current population; L(D) represents a random number; D represents the dimensionality; and α represents a random number following the standard normal distribution.

The ambush hunting strategy simulates the rapid dive behavior of a puffin after spotting prey. The displacement step size is adjusted using a velocity coefficient S to accelerate the convergence of individuals to high-quality solution regions, and the updated equations are shown in Equations (11) and (12).

(11) $$\vec{Z_{i}^{t+1}}=\vec{Y_{i}^{t+1}}*S$$

(12) S=tan((rand−0.5)∗π)

To balance search breadth and convergence speed, the algorithm merges the candidate positions generated in the two stages into a new solution set, sorts them according to fitness, and selects the top N individuals to form the next generation population, as shown in Equations (13)–(15).

(13) $$\vec{p_{i}^{t+1}}=\vec{Y_{i}^{t+1}}\cup \vec{Z_{i}^{t+1}}$$

(14) $$new=\mathrm{sort}\left(\vec{p_{i}^{t+1}}\right)$$

(15) $$\vec{X_{i}^{t+1}}=new(1:N)$$

#### 2.3.3. Underwater Foraging Phase

When the puffin enters the underwater foraging stage, its behavior shifts to a more refined search of the discovered high-value areas, emphasizing local development. This stage mainly includes three strategies.

(1) Collective Foraging simulates the puffin colony collaboratively surrounding schools of fish by moving toward the current best-performing individual. The position update is given by Equation (16).

(16) $$\vec{W_{i}^{t+1}}=\left\{\begin{array}{ll} \vec{X_{r1}^{t}}+F*L(D)*\left(\vec{X_{r2}^{t}}-\vec{X_{r3}^{t}}\right) & \mathrm{rand}\geq 0.5\\ \vec{X_{r1}^{t}}+F*\left(\vec{X_{r2}^{t}}-\vec{X_{r3}^{t}}\right) & \mathrm{rand}<0.5 \end{array}\right.\;$$

In this formula, F is the cooperation factor, which is primarily used to adjust the capture behavior of the polar seabirds. $r_{1},r_{2},r_{3}$ denote random numbers. The numbers fall within the range [1, N − 1]. $X_{r_{1}}^{t},X_{r_{2}}^{t},X_{r_{3}}^{t}$ represent the most optimal candidates randomly selected.

(2) The enhanced search occurs when the food is depleted in the current region. The seabird will change its position to conduct a deeper search. This strategy enables the algorithm to conduct more refined exploration near the optimal solution. The position update formula for this phase is given by Equations (17) and (18).

(17) $$\vec{Y_{i}^{t+1}}=\vec{W_{i}^{t+1}}*(1+f)$$

(18) $$f=0.1*(rand-1)*\frac{\left(T-t\right)}{T}$$

In this context, T is the overall number of iterations, and t represents the iteration count. f is the adaptive factor used to adjust the position of the polar seabird in the water.

(3) The predator-avoidance strategy simulates the polar seabird quickly moving to a safe zone when encountering danger. By introducing random perturbations, the algorithm can escape local optima. As shown in the Equation (19).

(19) $$\vec{Z_{i}^{t+1}}=\left\{\begin{array}{ll} \vec{X_{i}^{t}}+F*L(D)*\left(\vec{X_{r1}^{t}}-\vec{X_{r2}^{t}}\right) & \mathrm{rand}\geq 0.5\\ \vec{X_{i}^{t}}+\beta *\left(\vec{X_{r1}^{t}}-\vec{X_{r2}^{t}}\right) & \mathrm{rand}<0.5 \end{array}\right.$$

In the equation, β is a random number.

The candidate positions generated by these three strategies are merged, and the individual with the best fitness is selected to enter the next generation. The top N individuals are selected in order, as shown in Formulas (20)–(22).

(20) $$\vec{p_{i}^{t+1}}=\vec{W_{i}^{t+1}}\cup \vec{Y_{i}^{t+1}}\cup \vec{Z_{i}^{t+1}}$$

(21) $$new=\mathrm{sort}\left(\vec{p_{i}^{t+1}}\right)$$

(22) $$\vec{X_{i}^{t+1}}=new(1:N)$$

**Figure 7:**
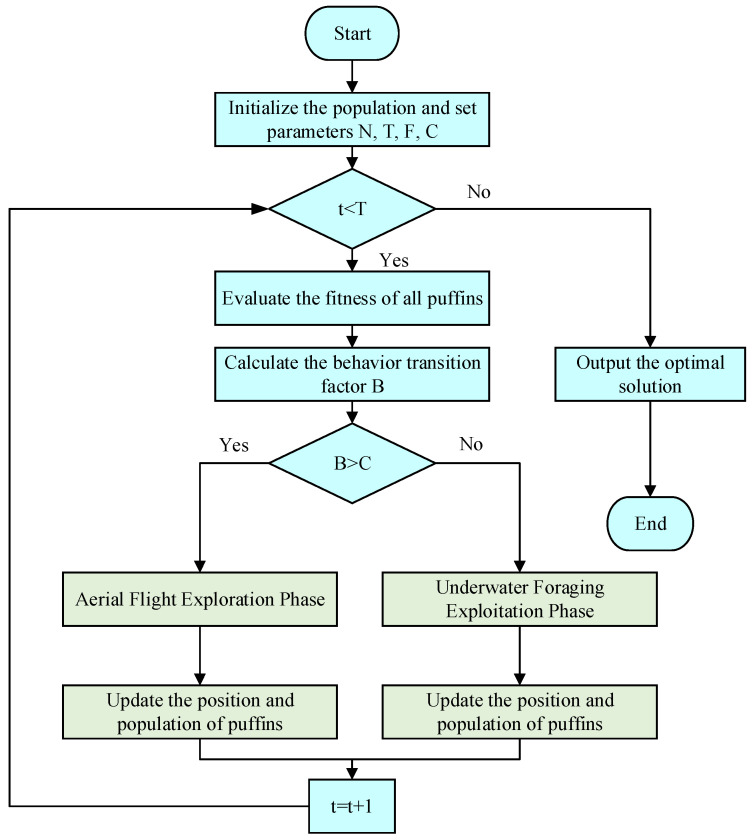
APO Algorithm Flowchart.

Figure 7 shows the overall process of the APO algorithm. This integrated conversion strategy enables the algorithm to consider multiple scenarios simultaneously and select the best position as the optimal solution.

#### 2.3.4. APO-TCN-LSTM-AM Model

**Figure 8:**
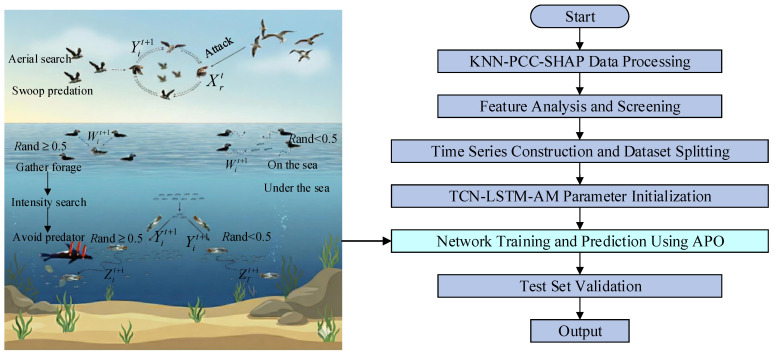
APO-TCN-LSTM-AM Method Diagram.

Although the TCN-LSTM-AM combined model achieves deep capture of temporal features in its structure, its predictive performance depends on the configuration of internal hyperparameters. Traditional methods, such as manual hyperparameter tuning, are not only inefficient when dealing with such vast parameter spaces but also struggle to find global optima. To address the challenge, this paper introduces the APO algorithm to build the APO-TCN-LSTM-AM prediction model, aimed at achieving adaptive optimization of key hyperparameters. The overall framework and optimization process of this method are shown in Figure 8.

First, the raw data are processed using KNN-PCC-SHAP. Through outlier imputation, linear and nonlinear correlation analysis, the feature subset with the highest information density is selected and divided into training, validation, and test sets in an 8:1:1 ratio, providing a high-quality data foundation for subsequent model training.

The hyperparameters to be optimized in the TCN-LSTM-AM model are defined as decision variables in the APO algorithm. The population size is set to N, and the maximum number of iterations is T. According to Equation (7), the Arctic puffin population X is initialized, where the position vector of each individual $X_{i}$ represents a set of potential hyperparameter combinations:

(23) $$X_{i}=[lr,K_{size},L_{tcn},N_{filters},L_{lstm},N_{units},P_{drop}]$$

In the formula, lr represents the learning rate, $K_{size}$ represents the TCN convolution kernel size, $L_{tcn}$ represents the number of TCN layers, $N_{filters}$ represents the number of TCN filters, $L_{lstm}$ represents the number of LSTM layers, $N_{units}$ represents the number of LSTM neurons, and $P_{drop}$ represents the Dropout rate.

To evaluate the merits of each set of hyperparameters, this paper uses the mean squared error (MSE) of the TCN-LSTM-AM model configured with the corresponding hyperparameters on the validation set as the fitness function of the APO algorithm. The smaller the MSE value, the higher the prediction accuracy of the model, and the higher the fitness of the corresponding puffin individual. The formula for calculating MSE is as follows:

(24) $$MSE=\frac{1}{n}\sum_{i=1}^{n}{\left(y_{i}-{\hat{y}}_{i}\right)}^{2}$$

In the formula, n is the total number of samples; $y_{i}$ is the actual value of PV power output, and ${\hat{y}}_{i}$ is the predicted value of PV power output.

In each iteration, the APO algorithm updates the position of the entire population by executing its unique aerial flight and underwater foraging strategies based on the fitness values of all individuals, thereby generating a new generation of potentially better hyperparameter combinations. When the aerial search condition is met, individuals simulate the aerial reconnaissance or ambush predation behavior of puffins, using Equations (8)–(12) to update their position in order to quickly locate potential areas in the vast solution space. When entering the underwater stage, individuals simulate collective foraging, enhanced search, or predator avoidance behavior, using Equations (16)–(19) to perform a refined search near the current optimal solution to improve the accuracy of the solution. Based on the candidate positions generated by different strategies, the individual with the best fitness is selected to enter the next generation according to Equations (20)–(22).

Finally, it is determined whether the number of iterations has reached the maximum value T. If not, the loop continues; if it has, the individual with the best fitness $X_{best}$ is output. The optimal combination of hyperparameters found is used to construct the final TCN-LSTM-AM model. This model will undergo final performance verification on the test set to objectively evaluate the prediction accuracy and generalization ability of the entire framework.

## 3. Results and Discussion

### 3.1. Experimental Setup

**Figure 9:**
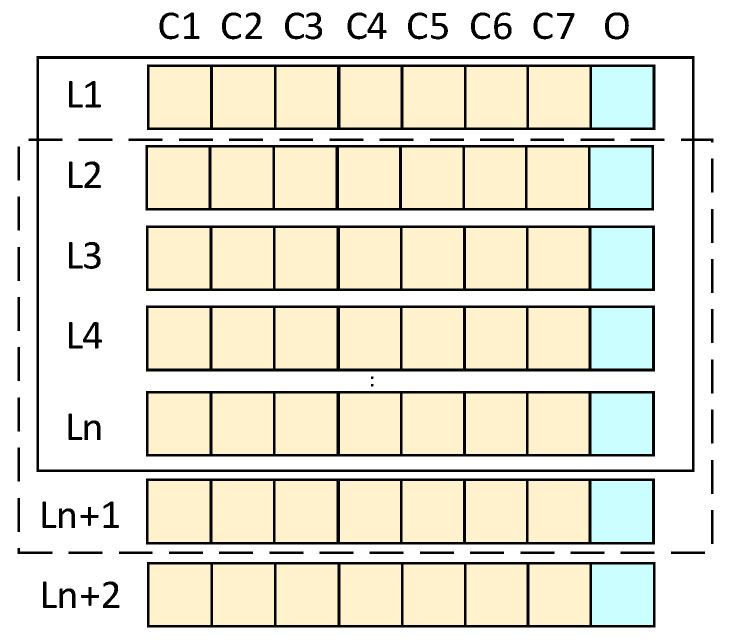
Schematic Diagram of the Sliding Window Principle.

The dataset used in this study comes from a PV power station in Xinjiang, Northwest China, and the time range is from 1 January 2019 to 31 December 2019. The data distribution information is shown in Appendix A Table A1. To ensure the model’s generalization capability, an open-source dataset [51] was subsequently introduced for supplementary validation. The dataset was divided into training, validation, and test sets at an 8:1:1 ratio. The model employed a rolling window prediction method, as illustrated in Figure 9, utilizing historical data from the preceding 24 time steps as input to forecast the PV power output for the next time step. This experiment is based on the Ubuntu 22.04 operating system and uses the Python 3.9 programming language for algorithm implementation and model training. The specific hardware and software environment configuration of the experimental platform is shown in Table 2.

To comprehensively evaluate the predictive performance of the model, this study selected five widely used error evaluation metrics, root mean square error (RMSE), coefficient of determination (R^2^), mean absolute error (MAE), mean absolute percentage error (MAPE), and normalized root mean square error (NRMSE), as defined in (25)–(29).

(25) $$MAE=\frac{1}{n}\sum_{i=1}^{n}{\left(y_{i}-{\hat{y}}_{i}\right)}^{2}$$

(26) $$RMSE=\sqrt{\frac{1}{n}\sum_{i=1}^{n}{\left(y_{i}-{\hat{y}}_{i}\right)}^{2}}$$

(27) $$R^{2}=1-\frac{\sum_{i=1}^{n}{\left(y_{i}-{\hat{y}}_{i}\right)}^{2}}{\sum_{i=1}^{n}{\left(y_{i}-{\bar{y}}_{i}\right)}^{2}}$$

(28) $$MAPE=\frac{1}{n}\sum_{i=1}^{n}\left|\frac{y_{i}-{\hat{y}}_{i}}{y_{i}}\right|\times 100\%$$

(29) $$NRMSE=\frac{\sqrt{\frac{1}{n}\sum_{i=1}^{n}{\left(y_{i}-{\hat{y}}_{i}\right)}^{2}}}{y_{max}-y_{min}}$$

In the formula, n is the total number of samples; $y_{i}$ is the actual value of PV power output, and ${\hat{y}}_{i}$ is the predicted value of PV power output.

### 3.2. Data Processing and Correlation Analysis

#### 3.2.1. KNN Processing

**Figure 10:**
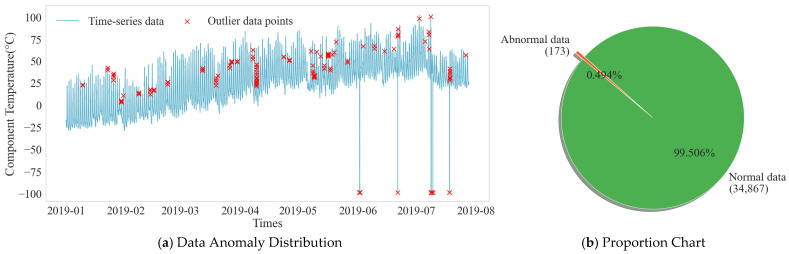
Data Anomaly Distribution and Proportion Chart.

In the process of photovoltaic data acquisition and transmission, outliers are inevitable, and these outliers can interfere with the training effect of the model. As shown in Figure 10, the temperature time-series distribution of the example component shows significant deviations from the normal fluctuation range. Anomaly detection was performed on the dataset, and the statistical results are shown in Figure 10. Out of 35,040 data points, 173 outliers were identified, accounting for approximately 0.494%. To ensure data quality, this paper uses the KNN algorithm to correct the outliers, leveraging their inherent relationship with multiple related features to generate reasonable replacement values. The data sequence after KNN interpolation regained its integrity, and the trend maintained good consistency with the periodicity and volatility of the original data.

#### 3.2.2. Feature Correlation Analysis

**Figure 11:**
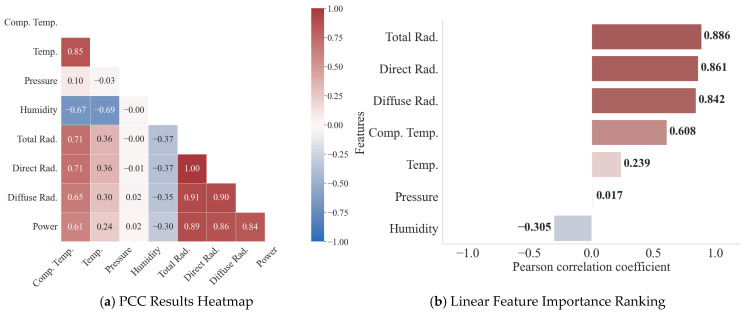
PCC Heatmap and Linear Feature Importance Ranking.

To assess the linear relationship between various meteorological features and actual PV power, and to initially reduce feature redundancy, this paper conducts a Pearson correlation analysis. The feature correlation heatmap and correlation coefficient ranking are shown in Figure 11. The input features exhibit a pronounced linear correlation with the PV power output. Solar radiation components emerge as the dominant drivers of power generation, with total (0.886), direct (0.867), and diffuse (0.842) radiation exhibiting pronounced positive coefficients. In contrast, humidity displays an inverse relationship (−0.305), while module and ambient temperatures show moderate positive associations of 0.608 and 0.239, respectively. However, the linear correlation between air pressure (0.017) and power is extremely weak.

#### 3.2.3. Feature Depth Selection Based on SHAP

Considering that PCC can only capture linear relationships, while the interaction and nonlinear effects between features are also quite important for model prediction, this paper further adopts the SHAP method to explore the nonlinear effects between features.

**Figure 12:**
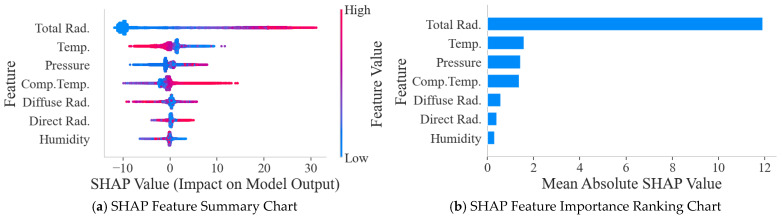
SHAP Feature Summary Chart and Feature Importance Ranking Chart.

As shown in Figure 12, total radiation is the most significant factor affecting power output, while temperature and diffuse radiation are also quite important. Although features such as relative humidity have a weaker linear correlation than irradiance, their SHAP values indicate that they still play a crucial role in the model’s complex decision-making. Total radiation, temperature, air pressure, and component temperature features have a significant impact on model predictions. Air pressure exhibits weaker correlation in linear analysis but also demonstrates significance in SHAP results.

Although diffuse radiation and direct radiation rank relatively high in importance, they were not included in the final feature subset. The main reason is that, physically speaking, total radiation is the sum of direct and diffuse radiation energy. Using all three as inputs simultaneously would introduce severe multicollinearity, leading to redundant feature information, which would not only increase model complexity but also potentially interfere with the model’s assessment of the independent contribution of each feature. Therefore, selecting the most representative and comprehensive total radiation as the sole irradiance input feature is a more efficient strategy, as verified in the SHAP ranking results.

The summary plot further elucidates the polarity and distributional characteristics of each feature’s contribution to the forecasting outcomes. Taking total radiation as an example, samples with higher eigenvalues generally correspond to higher positive SHAP values, indicating that total radiation has a strong positive feedback effect on power output. In contrast, air pressure exhibits a complex nonlinear relationship; regardless of whether its value is too high (red dots) or too low (blue dots), a large number of samples show negative SHAP values, indicating that excessively high or low air pressure can suppress power output.

Guided by these analytical insights, we isolated four pivotal variables—total radiation, temperature, pressure, and component temperature—to constitute the optimal input vector for the final forecasting model.

#### 3.2.4. Quantitative Validation of Feature Engineering Effectiveness

To validate the effectiveness of the KNN-PCC-SHAP feature engineering proposed in this paper, we designed four scenes for comparative experiments. The data processing methods and feature selection are detailed in Table 3. All scenes employed a baseline LSTM model as the predictor, with the baseline LSTM parameters for the experiments shown in Table 4.

**Figure 13:**
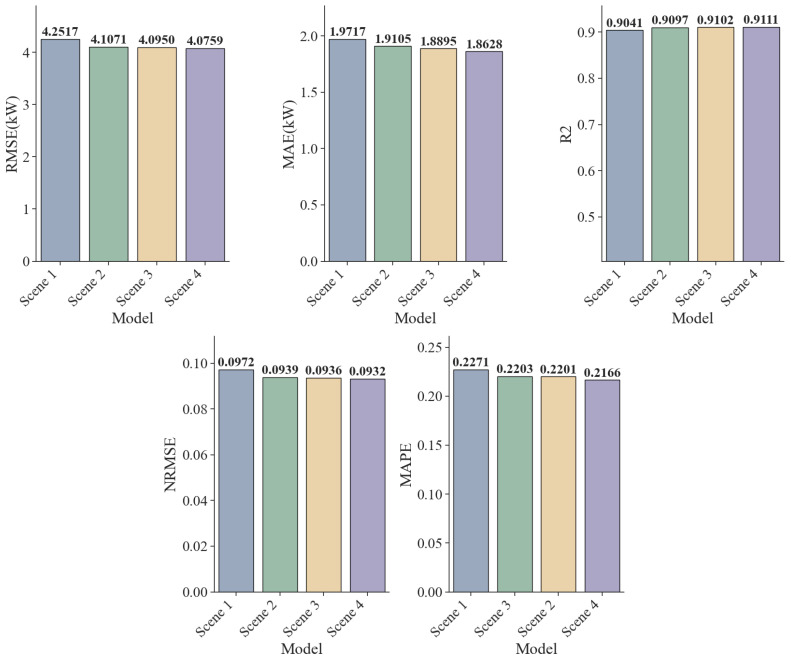
Comparison Chart of Errors for Each Indicator.

Table 5 and Figure 13 quantify the marginal contribution of each algorithm to model performance in feature engineering. We view this process as a series of progressive ablation experiments, and the results demonstrate the inherent improvement logic from data cleaning to feature selection.

In Scene 2, the KNN algorithm was used to clean outliers in the original data, resulting in a significant reduction in the model’s RMSE from 4.2517 kW to 4.1071 kW, a decrease of 3.4%. Furthermore, the normalized mean squared error (NRMSE) improved from 0.0972 to 0.0939. These results indicate that missing or outlier points in the original photovoltaic data severely disrupt the continuity of the time-series data, while the KNN method successfully repaired these structural breaks, effectively suppressing noise interference and providing a cleaner input distribution for model training.

Building on this, Scene 3 introduces the PCC method into the feature selection process. The improvement in various indicators was relatively moderate, demonstrating that removing redundant features linearly unrelated to the target variable can enhance predictive performance. Scene 2, using linear feature selection, minimizes the loss of key information and reduces the dimensionality of the feature space, thus mitigating the risk of overfitting and achieving a lightweight model.

Scene 4 further improves data input quality by introducing the SHAP method. In this scene, all model error metrics reach their lowest levels across the board: RMSE drops to 4.0759 kW, MAE to 1.8628 kW, MAPE is optimized to 0.2166, and R^2^ achieves a breakthrough of 0.9111. The results demonstrate the limitations of using only linear feature selection. The conversion of solar energy represents an intricate multi-physics coupling phenomenon, wherein climatic variables often exert nonlinear influences on the final power output. The SHAP method can uncover these crucial nonlinear interactions overlooked by PCC, compensating for the blind spots of linear methods and thereby improving the quality of model data input.

Experimental results demonstrate that the scientific validity of the KNN-PCC-SHAP framework is verified through data cleaning by KNN, linear noise reduction by PCC, and nonlinear feature selection by SHAP. Each step is indispensable, and together they construct a high-quality feature set with the highest information density.

### 3.3. Performance Comparison of Predictive Model Architectures

#### 3.3.1. Parameter Settings

The key parameter settings for the core model in this paper are as follows. The TCN layer count is set to 1 layer, the number of convolutional kernels is set to 64, the kernel size is set to 2, the stride is set to (1, 2, 4), and Dropout1 is set to 0.3; the LSTM layer count is set to 1 layer with 128 neurons, and Dropout2 is set to 0.3; The Dense layer is set to 1 layer. The ReLU activation function is used throughout the model. The model was trained for 50 iterations using the Adam optimizer with a learning rate of 0.0001. We adopted a batch size of 64 and minimized MAE as the objective function. All other parameters are set to default values. All models include a back-normalization step. To ensure the fairness of the comparative experiments, the parameter settings for the other models are consistent with those described above. The parameter settings for the other comparative models are shown in Table 6.

#### 3.3.2. Predictive Model Performance Comparison

For the performance of the proposed model to be comprehensively evaluated, a series of representative benchmark models should be selected for comparison. All models were trained using the optimal feature set processed through KNN-PCC-SHAP.

**Figure 14:**
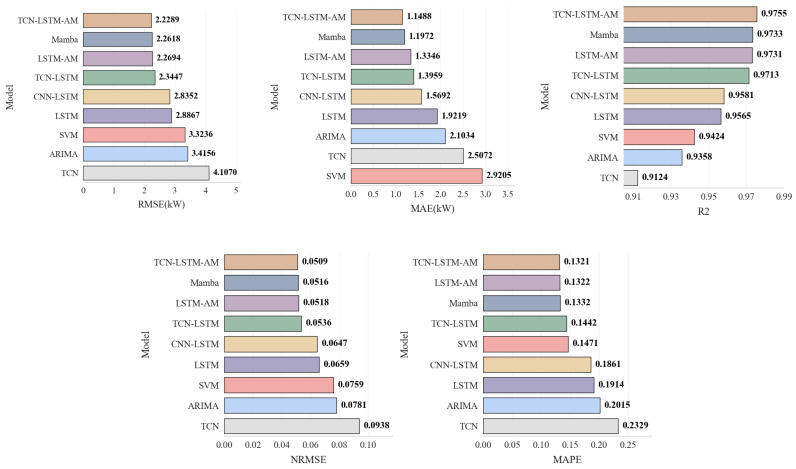
Performance Metrics Comparison Chart for Each Model.

Table 7 and Figure 14 show the performance comparison between the proposed model and various benchmark models on the test set. First, for the traditional statistical model ARIMA, its RMSE is 3.4156 kW. Although it outperforms the single TCN model in accuracy, its computational complexity is high, and training time is excessive in practical applications. Faced with high-frequency sampled photovoltaic time-series data, the differencing and parameter fitting processes of ARIMA are difficult to accelerate in parallel using GPUs, resulting in low model update efficiency and failing to meet the real-time requirements of photovoltaic systems.

In contrast, deep learning models demonstrate superior computational potential and feature capture capabilities when processing large-scale data. Among the basic models, LSTM significantly outperforms TCN and SVM, with an RMSE of 2.8867 kW, compared to 4.1070 kW for TCN and 3.3236 kW for SVM. This indicates that recurrent neural networks have a natural advantage in capturing long-term dependencies in photovoltaic sequences.

Combining TCN as a feature extractor with LSTM to construct the TCN-LSTM model reduces the RMSE to 2.3447 kW, a reduction of approximately 18.8% compared to the single LSTM model. This demonstrates that the ability of TCN to extract local detail features effectively complements the long-term memory mechanism of LSTM.

Introducing the AM further optimizes the model performance. Adding an attention layer significantly reduces the error for both LSTM and TCN-LSTM. The proposed TCN-LSTM-AM model integrates the advantages of all three, outperforming the state-of-the-art Mamba model (RMSE 2.2618 kW), with a further reduced RMSE of 2.2289 kW, an MAE of 1.1488 kW, and an R^2^ of 0.9755. Compared to the TCN-LSTM model, the RMSE was significantly reduced, while NRMSE and MAPE also reached the lowest levels of 0.0509 and 0.1321, respectively.

**Figure 15:**
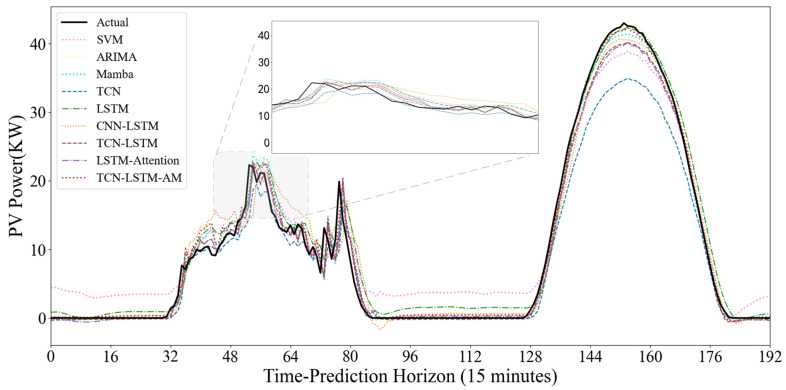
Non-Clear-Sky Scenario Prediction Curve.

**Figure 16:**
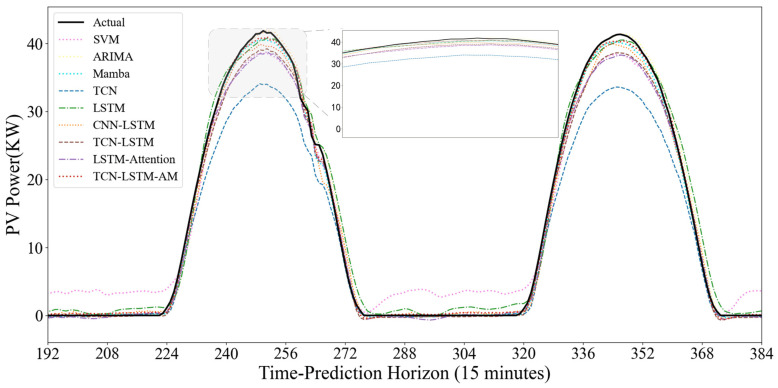
Clear-Sky Scenario Prediction Curve.

To evaluate the model’s dynamic prediction capability under different operating conditions, Figure 15 and Figure 16 show the prediction results under two typical weather conditions: sunny and non-sunny. In the sunny scenario where the power curve is smooth and highly regular, the SVM model shows good trend fitting during the day, but exhibits significant abnormal fluctuations at night. The noise during the nighttime period largely indicates that the SVM is overly sensitive to low-value ranges and has weak anti-interference capabilities. The TCN model shows severe systematic underestimation; the peak value of its predicted curve is far lower than the actual value. It is difficult to accurately predict the extreme values of PV power using only the convolutional structure, exhibiting a significant peak-shaving phenomenon. However, the TCN model is more suitable for feature extraction. Models such as ARIMA, Mamba, and LSTM generally have good fits and can closely follow the rising and falling trends of actual power, but there is still room for improvement in the fit at the peaks.

Traditional CNN models typically include pooling layers, which reduce the temporal resolution of time-series data, resulting in the loss of crucial temporal details. However, TCN employs dilated causal convolution, increasing the receptive field while ensuring no future information leakage during feature extraction. This allows TCN to capture local patterns and short-term fluctuations over a longer historical period with fewer layers, while maintaining consistency in the input and output time lengths.

In this study, while a single TCN excels at parallel feature extraction, it is less flexible than recurrent neural networks when handling long-term dependencies in long sequences. LSTM, while possessing strong memory capabilities, is susceptible to noise interference and has low training efficiency when dealing with high-dimensional, noisy raw photovoltaic data. Therefore, this model utilizes TCN as an efficient encoder to extract local high-frequency features and LSTM as a decoder to learn long-term temporal dependencies. This strategy of extracting first and then memorizing effectively reduces the learning difficulty of LSTM, allowing it to focus on capturing the diurnal periodic trend of PV power.

Standard TCN-LSTM models typically use the output of the last time step of the LSTM as the final prediction result. This is based on the fact that in actual PV power prediction, certain specific moments have a far greater impact on the future than other moments. TCN-LSTM-AM, by introducing an attention mechanism (AM), dynamically calculates similarity scores and assigns weights based on the relevance of input features to the prediction target. Through the mapping between queries (Q) and key-value pairs (K-V), the model can automatically identify and focus on historical hidden states that are more critical to the current prediction. This allows the model to ignore irrelevant interference and accurately focus on key factors causing power fluctuations when facing complex operating conditions, such as cloudy days, thus significantly reducing prediction errors. The TCN-LSTM-AM model outperforms similar hybrid architectures such as CNN-LSTM and TCN-LSTM in all metrics. Its performance advantage stems from the complementary functions of each component in processing the temporal characteristics of photovoltaic data.

The performance differences between models are more pronounced in cloudy days with variable weather and drastic power curve fluctuations. The SVM model consistently produces artificially high prediction values at night and during low-power periods, exhibiting excessive sensitivity to noise. While Mamba and ARIMA generally show normal trends, their predicted curves often lag behind actual values in the volatile time step range of 64–80, failing to respond promptly to sudden power drops caused by rapid cloud movement. The single TCN model continues its conservative prediction strategy, significantly underestimating power at the peaks of fluctuations. The CNN-LSTM model exhibits some instability, predicting significant negative values in the low-power range of 80–96, violating the physical laws of PV power generation and revealing its insufficient robustness in handling boundary conditions.

In contrast, the TCN-LSTM-AM model demonstrates superior dynamic tracking performance. In the most challenging time step range of 40–80, where actual power experienced multiple drops and rebounds, our model closely follows the black solid line with minimal lag. At night, the model quickly regresses and stabilizes near zero, without exhibiting SVM noise or CNN-LSTM outliers. The results demonstrate that the TCN-LSTM-AM architecture maintains accuracy during volatile periods and remains stable during nighttime.

### 3.4. Comparative Analysis of Algorithm Performance Optimization

#### 3.4.1. Experimental Design

To validate the superiority of the APO algorithm employed in this paper, it was compared with the genetic algorithm (GA), particle swarm optimization (PSO), and gray wolf optimization (GWO) algorithms. To ensure fairness, all optimization algorithms were applied to optimize the same TCN-LSTM-AM model architecture. All experiments used the same optimal feature subset as input. The fitness function for all algorithms was the model’s RMSE on the validation set, with the objective of minimizing it. To guarantee fairness, the key common parameter settings for all algorithms were kept consistent, as detailed in Table 8.

#### 3.4.2. Results and Analysis

To visually compare the optimization performance of different algorithms, we recorded the final prediction performance of the TCN-LSTM-AM model optimized using various algorithms on the test set. The optimal learning rate identified by this model is 1 × 10^−4^, with a TCN convolution kernel size of 3, 1 TCN layer, 64 TCN filters, 1 LSTM layer, 96 LSTM neurons, a dropout rate of 0.42, and a batch size of 128. The optimal parameter settings are shown in Table 9.

**Figure 17:**
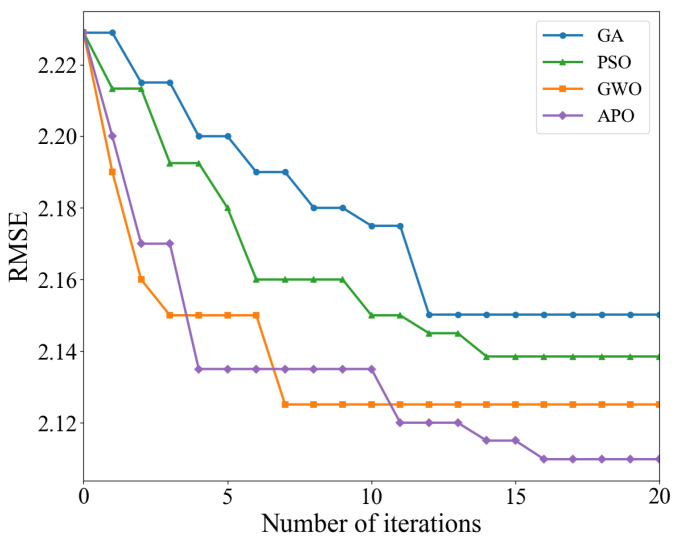
Convergence curve comparison.

Table 10 and Figure 17 provide a comparison of the quantization performance and dynamic convergence process of different metaheuristic algorithms when optimizing the same TCN-LSTM-AM model. With continuous algorithm evolution, GWO and APO typically outperform GA and PSO when handling the high-dimensional nonlinear parameter space of deep learning models. Although performance improvements in deep learning models often have marginal effects, APO still demonstrates significant fine-tuning capabilities. Compared to the mediocre GA, APO reduces RMSE by approximately 1.88%. Even compared to the suboptimal GWO algorithm, APO further reduces RMSE by approximately 0.72%. Furthermore, APO consistently demonstrates superiority in metrics measuring relative error and normalized error. Its NRMSE decreases to 0.0482, and MAPE decreases to 0.1272, both optimal values among all compared algorithms. This indicates that the APO-optimized model fits the overall trend better while maintaining better reliability across different power levels.

The iterative curves further reveal the dynamic optimization behavior of each algorithm under limited computational resources. APO and GWO show steeper curve slopes, indicating extremely high initial search efficiency. They can quickly locate the optimal region in the solution space within the first few iterations. In contrast, GA and PSO show relatively gentler declines, suggesting greater blindness in their early exploration and lower utilization of computational resources. This is attributed to APO’s mechanism of simulating puffin flight, which allows the population to make efficient global jumps across a large solution space, thus quickly narrowing the search range.

During the convergence phase, the GWO algorithm’s curve almost flattens out after about 6–8 iterations, possibly indicating a local optimum stagnation. Conversely, APO’s curve maintains a small but continuous downward trend during 10–20 iterations. This continuous optimization capability relies on APO’s proprietary underwater foraging strategy. When the algorithm enters a local region, the puffin’s refined search mechanism comes into play, enabling it to escape the local traps encountered by GWO and perform a more nuanced search for hyperparameters. This dynamic balance between exploration and exploitation is the core reason why the APO algorithm can find the global optimum.

In summary, in the complex high-dimensional hyperparameter optimization task of the TCN-LSTM-AM model, the APO algorithm not only comprehensively surpasses algorithms such as GA, PSO, and GWO in optimization accuracy, but also demonstrates advantages in convergence speed and the ability to avoid premature convergence. For the computationally expensive training of deep learning models, the APO algorithm, with its excellent balance between exploration and exploitation, can more effectively find hyperparameter configurations with better performance, achieving superior performance in fewer iterations, thus verifying its advanced nature and effectiveness in the application scenario presented in this paper.

### 3.5. Generalization Capability Validation Experiment

To verify the universality and robustness of the proposed prediction framework, this section introduces a public dataset for generalization capability verification. This dataset is from the operation data of a PV power station released by the State Grid Corporation of China in 2019–2020 [51], with a sampling interval of 15 min, including power output and related meteorological variables. The data are divided into training, validation, and test sets in an 8:1:1 ratio, and the prediction method is consistent with the aforementioned experiments.

After completing the KNN-PCC-SHAP feature engineering, the proposed APO-TCN-LSTM-AM model is compared with the benchmark model, and the results are shown in Table 11. From a single LSTM to TCN-LSTM, the RMSE decreased from 2.7821 kW to 2.6352 kW, and the MAE decreased significantly by 14.2%. This confirms that TCN, as an encoder, remains effective in extracting local features under new data conditions. After introducing AM, all indicators show slight but stable improvements, indicating that the model’s ability to focus on key time steps is universal across different sites. Finally, after APO hyperparameter optimization, the final model achieved good performance. Compared with the unoptimized TCN-LSTM-AM, the final model’s MAE decreased by approximately 13.3%, RMSE decreased by approximately 6.0%, and NRMSE dropped to a minimum of 0.0587. The APO algorithm possesses a powerful ability to find the global optimum in the unknown parameter space, significantly enhancing the model’s generalization performance.

**Figure 18:**
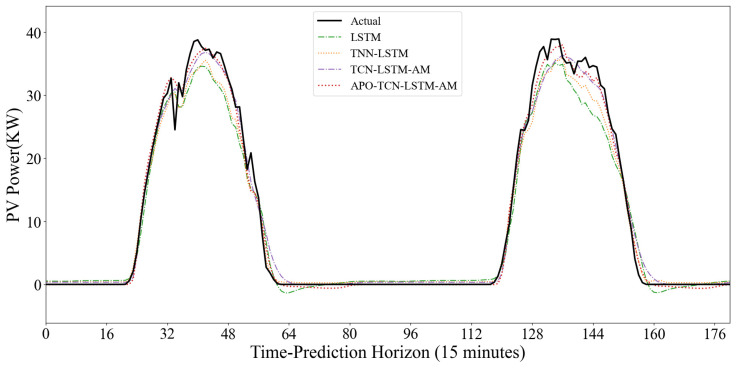
Comparison Chart of Typical Variable Weather Forecast Curves.

To further demonstrate the dynamic prediction performance of the model, two typical days with drastic weather fluctuations from the dataset were selected, and the predicted curves of each model were compared with the actual values. As shown in Figure 18, during periods of rapid changes in light intensity, the traditional LSTM model exhibits significant prediction lag, and significant errors occur when the power drops sharply to near 0, exposing its instability in handling boundary conditions. Although the TCN-LSTM model, with the introduction of TCN, outperforms the single LSTM in the overall trend and corrects some of the lag, it still exhibits significant amplitude deviation at the peaks of extremely rapid changes in light intensity, and its convergence speed in the low-power range is not fast enough. The APO-TCN-LSTM-AM model demonstrates superior dynamic tracking capabilities. Whether it is the rapid response during the power ramp-up phase or the smooth regression during the power descent phase, the model closely matches the actual power curve with very few noticeable abnormal fluctuations.

Experiments show that the proposed prediction framework maintains superior performance on publicly available datasets, with significantly reduced error indices and good dynamic fitting, proving that the method has strong generalization ability and potential for widespread application.

## 4. Conclusions

To address the challenges posed by the inherent volatility and intermittency of PV power generation to the stable operation of power systems, and the shortcomings of existing prediction methods in data processing, model construction, and parameter optimization, this paper proposes and verifies a high-precision PV power prediction framework integrating refined feature engineering, deep learning models, and intelligent optimization algorithms. Based on experiments and comparative analysis using local and public datasets, the main conclusions are as follows:

(1) The constructed KNN-PCC-SHAP feature engineering method can systematically solve problems such as data anomalies, feature redundancy, and nonlinear relationship mining. The results show that this method effectively improves the quality of input data by combining anomaly detection, linear filtering, and nonlinear selection, providing a more concentrated subset of features for subsequent models.
(2) The designed TCN-LSTM-AM prediction model fully combines the local feature extraction capability of TCN, the temporal memory characteristics of LSTM, and the focused attention mechanism of LSTM, overcoming the limitations of single models. Compared with various benchmark models, this model shows significant advantages in prediction accuracy, stability, and computational efficiency.
(3) The APO algorithm is used for model hyperparameter optimization. Compared with traditional algorithms such as GA and PSO, APO achieves a better balance between global search and local exploitation, and can find the optimal parameter combination of deep models more efficiently, thereby further improving prediction accuracy and generalization performance.

The proposed APO-TCN-LSTM-AM integrated framework achieved excellent prediction accuracy on real power plant datasets, with an RMSE of 2.1098 kW, NRMSE of 0.0482, MAE of 1.1073 kW, and R^2^ of 97.75%. Furthermore, this framework demonstrated strong generalization capabilities during validation on public datasets. This study confirms that deeply integrating refined data processing, advanced model architecture design, and intelligent algorithm optimization is an effective approach to improving PV power prediction accuracy. It provides reliable technical support for the precise scheduling and planning of PV power plants and offers a reference for prediction challenges in other renewable energy sources.

## Figures and Tables

**Table 1:** Summary of advanced models in recent years.

Reference (Year)	Methodology	Dataset	Performance Metrics	Key Contributions and Limitations
Ref [29] (2025)	TB-BTCGA	15 min	RMSE, MAE, R^2^	TB-BTCGA can capture the past and future, building long-term dependencies. However, it is not perfect in feature analysis.
Ref [30] (2024)	BiTCN-MixedSSM	5 min	RMSE, MAE, R^2^	Integrating BiTCN with hybrid SSM to capture dynamic temporal evolution is a complex approach, but adjusting the model’s structure is quite challenging.
Ref [31] (2025)	ISSA-LSTM	30 min	RMSE, MAE, R^2^	Adjusting the LSTM model based on ISSA yielded good prediction accuracy, but experimental comparisons with hybrid models are lacking.
Ref [32] (2024)	TCN-ECANet-GRU	5 min	RMSE, MAE, R^2^	TCN-ECANet-GRU shows good performance, but the model focuses on channel weighting and lacks in-depth exploration of feature interaction effects.
Ref [33] (2024)	ANN-GA/ANN-GWO	3 min	MSE, RMSE, MAE, R^2^	The article categorizes weather conditions and compares in detail the performance of GA and GWO in ANN structure optimization, but the model inputs are not rich enough.
Ref [34] (2024)	MLP-FGWO-PSO	15 min	MAE, MAPE, R^2^	The MLP-FGWO-PSO model proposed in this paper is well adapted to variable meteorological conditions, but lacks validation for its applicability to other terrains.
Ref [35] (2024)	CPO-CLA	Hourly	MSE, RMSE, MAE, R^2^	The paper applies the CPO algorithm to LSTM parameter optimization, constructing a CPO-CLA model with good results. However, its ability to capture long-term temporal patterns is limited.

**Table 2:** Experimental Platform Data.

Hardware and Software Environment	Main Attributes
Operating system	Ubuntu 22.04
CPU	12th Gen Intel Core i5-12500H @ 3.10 GHz
Memory	16 GB
Hard disk	512 SSD
Graphics card	NVIDIA GeForce RTX 4060
Software tools	Visual Studio Code
Algorithm environment	Python 3.9.21
Algorithm support framework	Tensorflow 2.10

**Table 3:** Summary of Mathematical Methods for Scene Parameters.

Scene	Method	Feature	Model
Scene 1	No action taken	Total Radiation, Direct Radiation, Diffuse Radiation, Component Temperature, Temperature, Pressure, Humidity	LSTM
Scene 2	KNN	Total Radiation, Direct Radiation, Diffuse Radiation, Component Temperature, Temperature, Pressure, Humidity	LSTM
Scene 3	KNN-PCC	Total Radiation, Direct Radiation, Diffuse Radiation, Component Temperature	LSTM
Scene 4	KNN-PCC-SHAP	Total Radiation, Temperature, Pressure, Component Temperature	LSTM

**Table 4:** Basic LSTM Parameters.

Parameter	Number
Learning rate	0.001
Number of LSTM layers	1
Number of LSTM neurons	64
Dropout	0.2
Loss function	MSE

**Table 5:** Error Comparison Data Across Scenes.

Scene	RMSE (kW)	MAE (kW)	R^2^	NRMSE	MAPE
Scene 1	4.2517	1.9717	0.9041	0.0896	0.2271
Scene 2	4.1071	1.9105	0.9097	0.0866	0.2201
Scene 3	4.0950	1.8895	0.9102	0.0863	0.2203
Scene 4	4.0759	1.8628	0.9111	0.0859	0.2166

**Table 6:** Model parameter settings.

Model	Parameter Settings
ARIMA	Order = (5, 1, 0)
SVM	Kernel = RBF, C = 1.0, Epsilon = 0.1
Mamba	Layers = 2, d_model = 64, d_state = 16, d_conv = 4, Expand = 2, max_iter = 100, learning_rate = 0.001, Dropout2 = 0.2
TCN	Layers = 2, Filters = 64, Kernel = 2, Dilations = [1, 2, 4, 8], max_iter = 100, learning_rate = 0.002, Dropout2 = 0.2
LSTM	Layers = 2, LSTM_Units = 128, max_iter = 100, learning_rate = 0.002, Dropout2 = 0.2
CNN-LSTM	Filters = 64, Kernel = 2, LSTM_Units = 128, max_iter = 100, learning_rate = 0.001, Dropout2 = 0.2
TCN-LSTM	Filters = 64, Kernel = 2, Dilations = [1, 2, 4, 8], LSTM_Units = 128, max_iter = 100, learning_rate = 0.0015, Dropout2 = 0.2
LSTM-AM	LSTM_Units = 128, max_iter = 100, learning_rate = 0.0012, Dropout2 = 0.2
TCN-LSTM-AM	Filters = 64, Kernel = 2, Dilations = [1, 2, 4, 8], LSTM_Units = 128, max_iter = 100, learning_rate = 0.0015, Dropout2 = 0.2

**Table 7:** Model Errors and Comparisons.

Model	RMSE (kW)	MAE (kW)	R2	NRMSE	MAPE
ARIMA	3.4156	2.1034	0.9358	0.0781	0.2015
SVM	3.3236	2.9205	0.9424	0.0759	0.1471
Mamba	2.2618	1.1972	0.9733	0.0516	0.1332
TCN	4.1070	2.5072	0.9124	0.0938	0.2329
LSTM	2.8867	1.9219	0.9565	0.0659	0.1914
CNN-LSTM	2.8352	1.5692	0.9581	0.0647	0.1861
TCN-LSTM	2.3447	1.3959	0.9713	0.0536	0.1442
LSTM-AM	2.2694	1.3346	0.9731	0.0518	0.1322
TCN-LSTM-AM	2.2289	1.1488	0.9755	0.0509	0.1321

**Table 8:** Parameter Settings.

Parameter	RMSE (kW)
Population size	25
Maximum number of iterations	20
Learning rate	[0.00001–0.005]
TCN convolution kernel size	[2–7]
Number of TCN layers	[1–3]
Number of TCN filters	[32–192]
Number of LSTM layers	[1–3]
Number of LSTM neurons	[32–320]
Dropout	[0.1–0.5]

**Table 9:** Optimal number of parameters.

Parameter	Number
Population Size	25
Maximum Number of Iterations	20
Learning Rate	0.00039
TCN Kernel Size	3
Number of TCN Layers	1
Number of TCN Filters	64
Number of LSTM Layers	1
Number of LSTM Neurons	96
Dropout	0.42

**Table 10:** Model Performance Comparison Under Different Optimization Algorithms.

Algorithm	RMSE (kW)	MAE (kW)	R^2^	NRMSE	MAPE
GA	2.1502	1.1354	0.9761	0.0491	0.1321
PSO	2.1385	1.1279	0.9765	0.0488	0.1316
GWO	2.1251	1.1186	0.9768	0.0485	0.1309
APO	2.1098	1.1073	0.9775	0.0482	0.1272

**Table 11:** Model Errors and Comparisons.

Model	RMSE (kW)	MAE (kW)	R^2^	NRMSE	MAPE
LSTM	2.7821	1.7226	0.9575	0.0664	0.1879
TCN-LSTM	2.6352	1.4785	0.9613	0.0629	0.1878
TCN-LSTM-AM	2.6199	1.3445	0.9623	0.0625	0.1853
APO-TCN- LSTM-AM	2.4619	1.1659	0.9649	0.0587	0.1659

## Data Availability

The original contributions presented in this study are included in the article. Further inquiries can be directed to the corresponding author.

## Appendix A

**Table A1:** Data distribution map of the Northwest Xinjiang region.

Feature	Count	Mean	Std	Min	25%	50%	75%	Max
Component Temperature	35,040.0	21.543158	24.128535	99.000	3.176252	23.0325	37.905000	102.202000
Temperature	35,040.0	11.324763	14.027540	18.662	−1.576500	13.1055	23.042250	40.131000
Pressure	35,040.0	927.778782	44.472451	99.000	926.032000	926.0630	933.795500	954.263000
Humidity	35,040.0	33.592505	20.761404	2.506	16.647000	28.1220	47.544250	94.921000
Total Radiation	35,040.0	245.602384	361.596645	99.000	0.000000	0.0000	429.875000	1677.220000
Direct Radiation	35,040.0	211.619020	319.596839	99.000	0.000000	0.0000	344.572000	1509.500000
Diffuse Radiation	35,040.0	111.402404	158.836518	99.000	0.000000	0.0000	209.204250	681.786000
Actual power	35,040.0	10.672173	14.918907	0.000	0.000000	0.0000	20.947835	49.309402
